# Implicit Theories of the Marital Institution and Partner Characteristics Preferences of Iranian Young Adults

**DOI:** 10.5334/irsp.1051

**Published:** 2025-12-08

**Authors:** Scott S. Hall, Hassan Shahi

**Affiliations:** 1 Ball State University, US; 2 Lorestan University, IR

**Keywords:** Implicit Theories, Marriage, Mate Preferences, Iran

## Abstract

Implicit Theories of the Marital Institution (ITMI) represent beliefs about whether marriage is a fixed or malleable relationship form. ITMI can influence moderate associations between related constructs such as marital beliefs and mate preferences. The current study tested for associations among certain beliefs about marriage (i.e., that roles should be gendered, that a marriage requires substantial effort), preferred characteristics in long-term mates (i.e., the KASER model of mate preferences), and ITMI. A sample of 588 young adults in Iran completed an anonymous survey focused on the constructs of interest. Gender was accounted for in all analyses given the relevance of gender in predominant mate-selection scholarship. Results indicated that the Fixed and Malleable ITMI were unexpectedly positively correlated. Regression analyses identified several interaction effects between marital beliefs and the preferred mate characteristics and a few marital beliefs by ITMI by gender interactions. Findings are explored in light of cultural context and implications for future research.

## Implicit Theories of the Marital Institution and Mate Preferences of Iranian Young Adults

Beliefs about marriage inform one’s intentions and decisions related to marriage (Hall 2006; Willoughby et al. 2015). For example, being more optimistic about marriage as a source of personal fulfillment could encourage a greater willingness to marry (Miles & Servaty-Seib 2010). Yet, what it actually means to be married—or to be a wife, husband, or spouse—can be somewhat subjective, particularly where cultural values elevate individuality (Cherlin 2004; Cherlin 2009). While ideas about the necessity, functions, and structure of marriage are somewhat shifting among younger generations (e.g., Barroso, Parker & Bennett 2020), marriage continues to be legal union with popular public recognition and cultural status throughout the world, with some basic shared understanding of the institution (Ortiz-Ospina & Roser 2020). Though some people seem to long for marriage because they perceive characteristics of marriage (e.g., lifetime commitment, sexual exclusivity) as desirable, others seem to avoid it because they perceive its characteristics as undesirable (Hall 2012a).

A specific type of marital belief targets the nature of marriage as a particular relationship form, namely, the extent to which marriage has clear, inherent qualities that one merely opts into or out of, and the extent to which marriage can be molded to suit one’s subjective preferences (Hall 2012a). Furthermore, this type of belief could interact with other marital beliefs to influence decisions or behavior related to marriage. For example, a woman who believes that marriage is too restrictive toward women, and who also believes that marriage has a clear, unchangeable set of parameters (i.e., ‘that’s just how marriage is’), might avoid getting married or enter marriage with some trepidation and defensiveness regarding expected restrictions. Conversely, if she believes that marriage is more flexible and can be molded to avoid the restrictiveness she perceives in other marriages, she might marry with confidence that her marriage can conform to her egalitarian ideals (Hall 2012b).

What people believe about the nature of marriage could potentially inform various attitudes and behaviors related to marriage, such as preferred characteristics in a life-long spouse. Studies show, for example, that heterosexual women have tended to highly value honesty and intelligence in a long-term partner whereas sexual attraction to signs of conventional masculinity mattered more for short-term relationships (Buss & Schmitt 1993; Buss & Schmitt 2019). This suggests that beliefs about what it means to be in a long-term relationship were relevant to choosing a partner. Beliefs about marriage, including the extent to which it has a rigid or flexible definition or parameters, might contribute to preferences about one’s eventual life partner or spouse. Such a possibility is the focus of the current study, conducted within the cultural context of the preferences of Iranian young adults.

### Implicit theories of the marital institution

Hall (2012a) proposed that beliefs about the flexibility of the meaning of marriage can be considered Implicit Theories of the Marital Institution (ITMI). The general concept of implicit theories has roots in the study of assumptions about intelligence—whether intelligence is thought of as a fixed trait or a malleable attribute that can be cultivated through effort (Dweck et al. 1995). This dichotomy was also applied to how people think about emotions (Tamir et al. 2007), sexuality (Bohns, Scholer & Rehman, 2015; Maxwell, et al. 2017), and romantic relationship formation (Franiuk, Cohen & Pomerantz, 2002; Knee 1998). Implicit theories have been shown to correspond with behavioral and psychological outcomes. For example, having a more malleable perspective on various characteristics has been associated with persistence during difficult learning tasks (Erdley & Dweck 1993; Sisk et al. 2018), better mental health (Tamir et al. 2007), the use of more active relationship coping skills (Knee 1998), and less positive feelings about ‘ghosting’ or abruptly cutting off a relationship without contact (Freedman et al. 2019).

ITMI is an application of the implicit theory framework toward differing beliefs about the changeable nature of marriage. Conceptually, fixed, and malleable theories about intelligence were thought to be distinct belief systems that could function independently. However, measurement has been more successful using a polarized single dimension because the malleable (or ‘incremental’) beliefs seemed too appealing to survey participants, creating problems with measurement variance (Dweck 1996). Other uses of the Implicit Theories framework have had more success measuring separate, often unrelated or negatively related dimensions, such as Implicit Theories of Relationships (Knee, Patrick & Lonsbary 2003) and Implicit Theories of Sexuality (Maxwell et al. 2017; see also Hall & Knox, 2024). Given the relational nature of the topic, the ITMI framework followed the lead of these latter studies and was formed with the expectation of having separate fixed and malleable dimensions (Hall 2012a). In the initial study, they appeared to function somewhat independently (modesty correlated at *r* = –.22, *p* < .001; Hall 2012b). Furthermore, ITMI tended to correspond with distinct perceptions. Hall (2012b) found that young adults with a more fixed ITMI tended to hold marital beliefs that were more oriented toward marriage being romantic, hierarchical, and sufficient for meeting companionship needs.

### Implicit theories as moderators

The role of implicit theories on various outcomes can be quite nuanced. Implicit theory beliefs can interact with other predictor variables, revealing a moderating role between independent and dependent variables. For example, Chinese high school students’ learning engagement was more closely associated with their past achievement when they had a more malleable rather than fixed view of intelligence (Li et al. 2017). Regarding the destiny of romantic relationships (a fixed perspective), greater relationship conflict was more strongly associated with having lesser relationship commitment for individuals who believed that relationships were destined to succeed or fail (Knee, Patrick & Lonsbary 2003). Similarly, endorsing a soulmate relationship theory (a fixed perspective) interacted with the perception of being with one’s soulmate, predicting higher relationship satisfaction for those who thought they were with their soulmate and lower relationship satisfaction otherwise (Franiuk, Cohen & Pomerantz 2002). Implicit theories could be thought of as having the ability to dampen or amplify the effect of other beliefs, attitudes, or perspectives that contribute to individual and relationship patterns and outcomes.

Following precedent, Hall (2012a) speculated that ITMI could also have a moderating effect on relevant constructs. Hall found that young adults that endorsed the fixed ITMI were more likely to want to marry when they also reported higher parental marital quality. Also, endorsing the fixed theory was associated with not wanting to marry when young adults viewed marriage as not having special or sacred status (i.e., just one of many types of relationships, or just a piece of paper). Findings were consistent with the idea that a fixed perspective might correspond with a tendency to assume that perceived desirable or undesirable aspects of marriage would inherently be a part of one’s own marriage and thus be salient to one’s own plans.

### The current study

Based on the premise that the meanings marriage holds for individuals inform intentions and decisions related to getting or being married (Hall 2006; Willoughby et al. 2015), two marital beliefs were studied that focus on day-to-day elements of marriage, or representing ‘marital processes’ beliefs dimension according to Marital Paradigm Theory (Willoughby et al. 2015; Willoughby & Hall 2015): Marital Roles (i.e., marriage functions best with traditional, gendered roles), and Marital Effort (i.e., a successful marriage requires great effort to achieve). These two marital beliefs have been shown to correspond with various individual characteristics and marital attitudes (e.g., Hall & Willoughby 2018; Shahi et al. 2023a; Willoughby & Hall 2015).

The outcome of interest is desired characteristics in a life partner or spouse. Foundational to much mate selection theory is the idea that people’s mate preferences are not random or arbitrary and are influenced at least in part by the objectives or functions of the relationship—to share recreational experiences, solidify economic security or social status, sooth insecurities, or satisfy subconscious biological drives, for example (Brumbaugh, Baren & Agishtein 2014; Buss 1995; Buss 2019; Conroy-Beam & Buss 2021; Gonzaga, Campos & Bradbury 2007). Relationship functions can differ based on relationship type. Indeed, substantive research indicates that people’s mate preferences differ based on whether they are seeking a short- or long-term relationship (Buss & Schmitt 1993; Buss & Schmitt 2019; Muggleton & Fincher 2017; Puts, Jones & DeBruine 2012). Similarly, spouses, compared to dating partners, have been found to share more similar characteristics with one another (Blackwell & Lichter 2004). Speculating with ITMI, a particular partner characteristic, such as one that signals high potential for wealth, especially might be desirable for someone who believes that a primary and inherent (i.e., fixed) function of marriage is to cultivate financial security.

Partner characteristic preferences can be operationalized and investigated in a variety of ways. The KASER model of long-term mate preferences presumes that ‘Humans prefer certain characteristics in choosing a long-term partner and such preferences in mate selection have solved sex differentiated adaptive problems in the deep evolutionary history of humans (homo sapiens)’ (Atari 2017, p. 1). The KASER measure or such characteristics was developed using qualitative and quantitative methods with Iranian samples. Five dimensions were identified and operationalized as such: Kindness/Dependability (‘K’; kind and understanding, honest, dependable, loyal, emotionally stable), Attractiveness/Sexuality (‘A’; physically fit, sex appeal, good looking), Status/Resources (‘S’; high income, financial prospects, favorable social standing), Education/Intelligence (‘E’; college graduate, intelligence), and Religiosity/Chastity (‘R’; religious, virginity). The current study used all the KASER dimensions as an exploration of possible connections among marital beliefs, ITMI, and mate preferences.

Extensive scientific research across various cultures generally indicates that men and women as groups somewhat diverge in their mate characteristic preferences, especially regarding long-term relationships (Bech-Sørensen & Pollet 2016; Buss 1989; Buss 1995; Eastwick & Finkel 2008). Typically, men more highly preference indicators of youth and fertility while women more highly preference indicators of status and economic provision. The KASER model has been used in multiple Middle Eastern countries to likewise identify gender differences (e.g., men placed more value on Attractiveness/Sexuality and women placed more value on Kindness/Dependability, Status/Resources, and Education/Intelligence; Atari et al. 2020). Thus, for the current study, gender differences were tested through interactions analyses with marital beliefs, ITMI, and with the interaction of marital beliefs and ITMI. Background factors of age, education level, and religiousness have also been found to associate with mate-selection processes (e.g., Fieder & Huber 2022; McClendon 2018; Schwarz & Hassebrauck 2012; Shahi et al. 2023b) and were treated as control variables in the current study.

The cultural context for the current study is also noteworthy. The research was conducted in Iran with a native Iranian sample. Predominantly Muslim countries like Iran are typically characterized by strong cultural values of embeddedness and hierarchy (Delkhamoush 2009), favoring conservation (security, conformity, and tradition) over openness to change (self-direction and stimulation), and favoring self-transcendence (benevolence and universalism) over self-enhancement values (hedonism, achievement, and power; Schwartz 2012; Schwartz & Sagiv 1995). Thus, views of marriage in Iran could skew toward a rigid perspective of marital meaning, with a strong emphasis on traditional roles and devotion. Furthermore, the ‘Qeirat’ values common in Iranian culture—which encapsules concepts such as honor, protecting a loved one, benevolence, respect, and sexual modesty (Atari, Chaudhary & Al-Shawaf 2020)—accentuate gendered ideals and active commitment related to marriage and family (Atari et al. 2017). While the current study makes no cross-cultural comparisons of ITMI or relationship beliefs, it was anticipated that the Iranian context could contribute to unique perspectives related to beliefs about marital roles and effort and should be considered when interpreting findings.

The purposes of the current study were to 1) explore the ITMI framework within a non-Western (historically under-studies) cultural context to see if and how the two ITMI dimensions emerge and relate to one another, and 2) explore how ITMI associate with partner characteristic preferences as direct predictors (main effects) and as potential moderators (interaction effects) of how marital beliefs associated with such preferences. Findings have implications for testing and refining the theoretical foundation of ITMI, illustrating how beliefs about aspects of marriage might contribute to partner characteristic preferences, and how beliefs about the nature of the flexibility of the marital institution might play a role in how other beliefs about marriage inform these preferences. Research that focuses on the implications of marital beliefs on preferences and intentions related to romantic relationships and marriage might oversimplify identified associations without attending to other assumptions about marriage to be redefinable. While someone might have a default belief about marriage that seemingly leads to certain behavior, the influence of that belief might be lessened if that person also believes that marriage is highly changeable and therefore the belief is more akin to a suggestion than a rule. Ultimately the findings can advance understanding of cognitive processes that inform intentions and decisions related to getting and being married. Selecting a life partner (for those who do) is a key decision with profound consequences for individuals and families, and understanding what influences this decision can inform efforts to promote among young adults’ positive relationship development and eventual individual and family flourishing.

## Method

### Procedure and participants

Students from campuses associated with a large university system located in a northwest region of Iran were invited to participate in extensive research related to romantic relationships (Authors, blinded). The ethics committee of the University approved the study which was conducted in accordance with the rules laid down in the seventh and current edition (2013) of the Declaration of Helsinki. Participation was voluntary and without compensation. Those included in the study must have been at least 18 years old at the time and unmarried. Participants (*N* = 644) completed a paper-and-pencil questionnaire. Students who did not report their gender (*n* = 10) or were married or divorced (*n* = 26) were removed from the analyses to focus on marital beliefs among unmarried women and men. The final sample included 588 participants with complete data on all the variables of interest, 49% of whom were females and 51% of whom were males. The mean age of the sample was 22.10 years (*SD* = 2.90). Nearly 3% of the participants were earning an associate’s degree, 91.7 were earning a bachelor’s degree, 4% were earning a master’s degree, and 0.3% were earning a doctorate degree. See Table 1 for descriptive data on all measures.

**Table 1:** Descriptive Statistics for all Variables (*N* = 588).


	%	*M* (*SD*)

Males	51	

Females	49	

Age		22.10 (2.90)

Religiosity		5.14 (2.67)

Education (earning)		

Associates	2.9	

Bachelors	91.7	

Masters	47.0	

Doctorate	0.3	

Marital Roles		3.61 (1.18)

Marital Effort		3.45 (.80)

Fixed IMTI		2.91 (.77)

Malleable ITMI		3.05 (.76)

Kindness/Dependability (K)		3.80 (.32)

Attractiveness/Sexuality (A)		3.04 (.70)

Status/Resources (S)		2.92 (.79)

Education/Intelligence (E)		3.09 (.75)

Religiosity/Chastity (R)		3.15 (.69)


### Measures

#### Sociodemographic variables

*Age* was recorded in years. *Education* (current degree being sought) was coded as 1 = associate’s degree, 2 = bachelor’s, 3 = master’s, 4 = MD, and 5 = PhD. *Religiousness* was measured on a single item on a scale from 1 to 10 with higher scores meaning greater religiousness: ‘To what extent do you consider yourself a religious person?’

#### ITMI

Measures of ITMI and marital paradigm beliefs from studies used in the Unites States were translated into Persian through the double translation method (English to Persian, then Persian to English, and again English to Persian and matching the two Persian translations). *Implicit Theories of the Marital Institution* (ITMI) were measured using seven items (Hall 2012b). Each item was a statement about what the participant ‘believe[s] about marriage,’ along a 5-point scale ranging from ‘strongly disagree’ to ‘strongly agree.’ Details about measure construction and reliability are reported in the results section.

#### Marital paradigm beliefs

Two types of marital beliefs were included as key marital process beliefs within a person’s marital paradigm (see Willoughby & Hall 2015). *Marital Roles* was measured with 3 items focusing on gendered roles (e.g., ‘Wives should have most of the say with decisions about housework and childcare,’ α = .71), with higher scores representing a traditional, hierarchical marital role structure. *Marital Effort* was measured with two items regarding the effort required to have a successful marriage (2 items) (e.g., ‘Happy marriages require hard work,’ α = .80), with higher scores representing greater effort.

#### Iranian mate preferences scale (IMPS)

The Iranian Mate Preferences Scale-20 (Atari 2017) is an abbreviated version of the KASER model mate preferences. The KASER model was developed based on qualitative interviews and quantitative testing with Iranian adults. Participants responded to 20 items that addressed the characteristics they would want in a spouse, which includes five different dimensions: *Kindness/Dependability* (8 items, *α* = .86), *Attractiveness/Sexuality* (3 items, *α* = .86), *Status/Resources* (3 items, *α* = .77), *Education/Intelligence* (3 items, *α* = .79), and *Religiosity/Chastity* (3 items, *α* = .71). Each item is rated on a 4-point scale: 1 (unimportant), 2 (slightly important), 3 (important), and 4 (very important).

### Results

#### Exploring ITMI in Iran

As with the original study (Hall 2012b), the seven items were analyzed in a principle-components factor analysis with varimax rotation confirmed a two-factor structure with the first factor having an Eigenvalue of 2.44 (34.91% of variance) and the second and Eigenvalue of 1.73 (24.73% of variance). Each item loaded on one of two factors with a score of .60 or greater. The first factor became the Fixed ITMI subscale and consists of the following four items: (a) ‘When you marry, you pretty much agree to become a certain kind of person (like others who are married),’ (b) ‘All happy marriages are pretty much the same,’ (c) ‘All long-term marriages have the same general characteristics,’ and (d) ‘There is a best way for married couples to organize their marriages’ (α = .75). The second factor became the Malleable ITMI subscale and consists of the following three items: (a) ‘It is up to the individuals who get married to decide what marriage should require of them,’ (b) ‘A marriage is whatever a particular couple decides marriage should be like for them,’ and (c) ‘Marriage can be whatever I want it to be’ (α = .63). This pattern mirrors that of the original findings with a US sample (Hall 2012b).

A bivariate Pearson correlation between the Fixed ITMI and the Malleable ITMI showed that they were (unexpectedly) positively correlated (*r* = .33, *p* < .001). As seen in Table 1, the Malleable ITMI was slightly more strongly endorsed than the Fixed ITMI, and both were above the midrange score of 2.5 for the measure. *T*-tests indicated no gender difference for endorsing the Fixed ITMI (*t*(586) = .93, *p* = NS) but that women scored significantly higher than men on the Malleable ITMI (*t*(586) = 1.82), *p* < .05). Correlations among all study variables are presented in Table 2.

**Table 2:** Bivariate correlation coefficients among background variables, marital beliefs, and KASER mate characteristics (*N* = 588).


	1	2	3	4	5	6	7	8	9	10	11	12

1. Religiousness	–											

2. Age	–.02	–										

3. Education	.03	.19***	–									

4. Marital Roles	.06	.06	.03	–								

5. Marital Effort	–.03	–.07	–.03	.09*	–							

6. Fixed ITMI	–.04	.06	.09*	.11**	.09*	–						

7. Mall. ITMI	–.05	–.01	–.03	–.15***	.03	.33***	–					

8. K	–.03	–.05	–.12**	.01	–.04	.06	–.01	–				

9. A	–.08	–.02	–.03	.17***	–.02	–.05	.02	.21***	–			

10. S	–.04	–.10*	–.07	–.02	–.04	–.02	–.02	.21***	.35***	–		

11. E	–.09*	–.08*	.04	.00	.00	.03	.00	.28***	.32***	.57***	–	

12. R	.22***	–.04	–.02	.26***	–.06	.12**	–.09*	.37***	.31***	.29***	.36***	–


*p < .05, **p < .01, ***p < 001.

## ITMI and partner characteristics preferences

**Figure 1:**
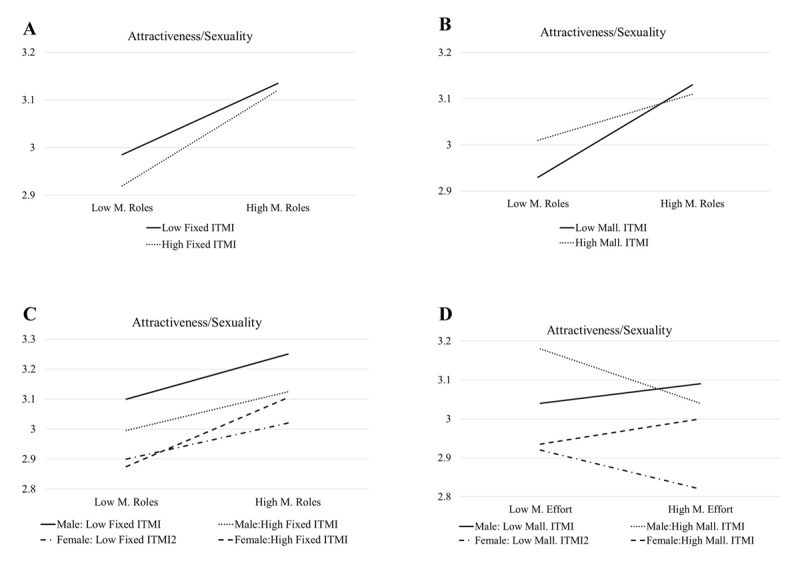
ITMI By Marital Belief By (When Applicable) Gender Predicting Attractivness/Sexuality.

Multiple Regression analyses indicated that ITMI were somewhat relevant to partner characteristics preferences, typically as moderating variables. Specifically, control variables (gender, religiousness, age, and education), marital beliefs (roles and effort), the ITMI, marital beliefs by gender interactions, ITMI by gender interactions, marital beliefs by ITMI interactions, and marital beliefs by ITMI by gender interactions were regressed on each of the KASER mate preferences dimensions (Table 3). For Kindness/Dependability, the model was significant (*F*(20, 567) = 1.72, *p* < .05) but ITMI did not have any significant main or interaction effects. For Attractiveness/Sexuality (A), the model was significant (*F* (20, 567) = 2.81, *p* < .001) and ITMI had no significant main effects but had several significant interaction effects. First, the Fixed ITMI interacted with Marital Role beliefs. A simple slopes interaction analysis that used regressions with adjusted Fixed ITMI to one standard deviation above and to one standard deviation below the mean indicated that the unstandardized coefficient for Marital Role beliefs at the higher and lower Fixed ITMI levels were .17 (*p* <.001) and .09 (*p* = .053) respectively. As illustrated in Figure 1a, this indicates that endorsing Marital Role beliefs was more positively correlated with desiring someone with higher levels of Attractiveness/Sexuality for those who also had higher fixed beliefs about marriage. Second, the Malleable ITMI interacted with Marital Role beliefs. The simple slopes interaction analysis (same procedures as described above but with Malleable ITMI) indicated that the unstandardized coefficient for Marital Role beliefs at the higher and Malleable ITMI levels were .08 (*p* = .059) and.17 (*p* < .001) respectively. As illustrated in Figure 1b, this indicates that endorsing Marital Role beliefs was more strongly, positively correlated with desiring someone with higher levels of Attractiveness/Sexuality for those who also had lesser malleable beliefs about marriage.

**Table 3:** Regression Coefficients for Variables Regressed on Each KASER Mate Characteristic (*N* = 588).


	KINDNESS/DEPENDABILITY (K)			ATTRACTIVENESS/SEXUALITY (A)			STATUS/RESOURCES (S)			EDUCATION/INTELLIGENCE (E)			RELIGIOSITY/CHASTITY (R)		
				
	*B*	*SE B*	*β*	*B*	*SE B*	*β*	*B*	*SE B*	*β*	*B*	*SE B*	*β*	*B*	*SE B*	*β*

Gender (Male = 1)	–.07	.03	–.10*	.15	.06	.11**	–.74	.06	–.47***	–.36	.06	–.24***	–.07	.06	–.05

Religiousness	.00	.00	–.02	–.01	.01	–.05	–.01	.01	–.03	–.02	.01	–.08	.06	.01	.23***

Age	–.01	.00	–.07	–.01	.01	–.05	–.02	.01	–.08*	–.03	.01	–.10*	–.02	.01	–.07

Education	–.11	.04	–.13***	–.04	.08	–.02	–.08	.08	–.04	.13	.08	.06	–.06	.07	–.04

Marital Roles (MR)	–.01	.02	–.03	.14	.04	.24***	.04	.04	.06	.05	.04	.07	.14	.04	.23***

Marital Effort (ME)	–.02	.02	–.05	–.04	.05	–.05	–.02	.05	–.02	–.01	.05	–.02	–.11	.05	–.13*

Fixed ITMI (FITMI)	.04	.03	.10	.00	.06	.00	.11	.06	.10	.12	.06	.12	.22	.06	.25***

Mall. ITMI (MITMI)	–.04	.03	–.10	.01	.06	.01	–.04	.06	–.04	–.01	.06	–.01	–.17	.06	–.19***

MR*Gender	.03	.02	.08	–.04	.05	–.05	.12	.05	.12*	.01	.06	.01	.04	.05	.04

ME*Gender	–.02	.03	–.03	–.01	.07	–.01	–.02	.07	–.01	.02	.08	.02	.05	.07	.04

FITMI*Gender	.00	.04	–.01	–.07	.09	–.05	–.14	.09	–.10	–.10	.10	–.08	–.16	.08	–.14*

MITMI*Gender	.03	.04	.05	.08	.09	.06	–.14	.09	–.09	–.07	.10	–.05	.09	.09	.06

MR*FITMI	.01	.02	.03	.12	.04	.19***	.07	.04	.10	.04	.04	.05	.08	.04	.12*

MR*MITMI	–.02	.02	–.06	–.08	.04	–.13*	–.04	.04	–.06	–.09	.04	–.13*	–.07	.03	–.11*

ME*FITMI	.04	.03	.07	.04	.07	.03	.05	.08	.04	.09	.08	.07	.07	.07	.06

ME*MITMI	.01	.03	.03	.10	.06	.09	–.01	.07	.00	.16	.07	.13*	.04	.06	.04

MR*FITMI*Gender	.03	.03	.07	–.17	.07	–.19**	.00	.07	.00	–.02	.07	–.02	.02	.06	.02

MR*MITMI*Gender	–.02	.03	–.05	.07	.07	.07	.05	.07	.05	.04	.07	.04	.02	.06	.02

ME*FITMI*Gender	–.04	.05	–.06	–.04	.10	–.02	–.20	.10	–.11	–.13	.11	–.08	–.18	.09	–.12*

ME*MITMI*Gender	.01	.04	.01	–.19	.09	–.12*	–.05	.09	–.03	–.21	.10	–.13*	.04	.09	.02

R^2^	.06*			.09***			.26***			.11***			.17***		


*p < .05, **p < .01, ***p < 001.

Two three-way interactions were also identified. First, the fixed ITMI by Marital Roles by gender interaction was statistically significant. Simple slopes interaction analysis indicated that the unstandardized coefficient for Marital Role beliefs at the higher Fixed ITMI level was .07 (*p* = .26) for males and .24 (*p* < .001) for females, and at the lower level was .14 (*p* < .05) for males and .05 (*p* = .20) for females. As illustrated in Figure 1c, males and females who more strongly endorsed Marital Roles more highly valued Attractiveness/Sexuality, and females higher on Fixed ITMI had the strongest association between Marital Roles and Attractiveness/Sexuality. Second, the Malleable ITMI by Marital Effort by gender interaction was statistically significant. The simple slopes interaction analysis indicated that the unstandardized coefficient for Marital Effort beliefs at the higher Malleable ITMI level was –.12 (*p* = .06) for males and .03 (*p* = .61) for females, and at the lower level was .02 (*p* < .83) for males and –.11 (*p* = .13) for females. As illustrated in Figure 1d, males with higher Malleable ITMI and females with lower ITMI had a negative association between Marital Effort and Attractiveness/Sexuality, while males with lower Malleable ITMI and females with higher Malleable ITMI had a slight positive association between Marital Effort and Attractiveness/Sexuality.

**Figure 2:**
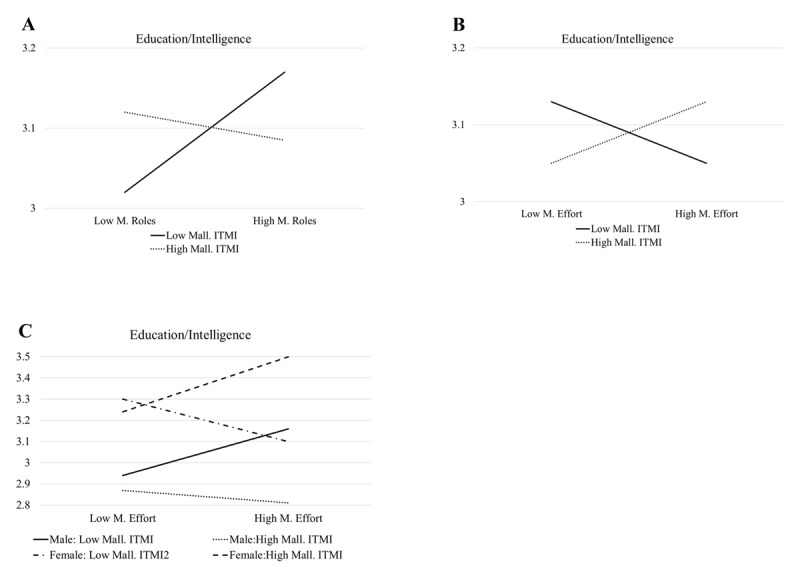
ITMI By Marital Belief By (When Applicable) Gender Predicting Education/Intelligence.

For Status/Resources (S), the model was significant (*F* (20, 567) = 3.39, *p* < .001) and ITMI had no significant main effects or interaction effects. Of note, Marital Roles interacted with gender to predict Status/Resources, with separate regressions indicating a positive association between Marital Roles (.17, *p* < .001) for males and no association for females (.01, *p* < .78). For Education/Intelligence, the model was significant (*F* (20, 567) = 9.87, *p* < .001) and ITMI had no significant main effects but had several significant interaction effects. First, the Malleable ITMI interacted with Marital Role beliefs. The simple slopes interaction analysis indicated that the unstandardized coefficient for Marital Role beliefs at the higher and lower Malleable ITMI levels were –.00 (*p* = .96) and .10 (*p* < .05) respectively. As illustrated in Figure 2a, this indicates that endorsing Marital Role beliefs was positively correlated with desiring someone with higher levels of Education/Intelligence for those with lower Malleable ITMI and slightly negatively associated for those with higher Malleable ITMI. Second, the Malleable ITMI interacted with Marital Effort beliefs. The simple slopes interaction analysis indicated that the unstandardized coefficient for Marital Role beliefs at the higher and lower Malleable ITMI levels were .03 (*p* = .62) and –.04 (*p* = .55) respectively. As illustrated in Figure 2b, this indicates that endorsing Marital Effort beliefs was positively correlated with desiring someone with higher levels of Education/Intelligence for those with higher Malleable ITMI and negatively correlated with Lower Malleable ITMI. Finally, a three-way interaction was also identified for Fixed ITMI by Marital Roles by gender. Simple slopes interaction analysis indicated that the unstandardized coefficient for Marital Effort beliefs at the higher Malleable ITMI level was –.04 (*p* = .62) for males and .10 (*p* = .13) for females, and at the lower level was .05 (*p* = .56) for males and –.14 (*p* = .06) for females. As illustrated in Figure 2c, males and females who more strongly endorsed Marital Effort also valued Education/Intelligence more highly, and females (especially) higher on Malleable and males lower on Malleable had positive association between Marital Effort beliefs and Education/Intelligence.

**Figure 3:**
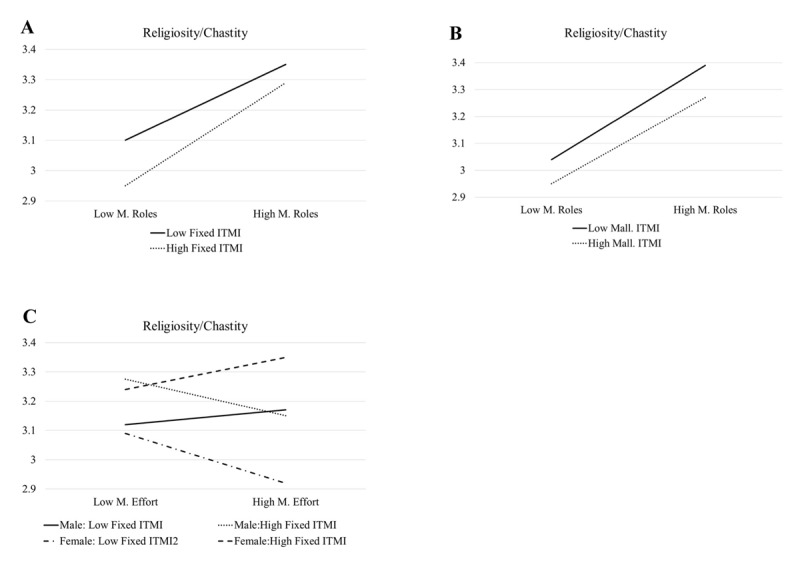
ITMI By Marital Belief By (When Applicable) Gender Predicting Religiosity/Charity.

For Religiosity/Chastity (R), the model was significant (*F* (20, 567) = 5.94, *p* < .001) and Fixed ITMI had a positive main effect and Malleable ITMI had negative main effect. Three slight interactions were statistically significant. First, the Fixed ITMI interacted with Marital Role beliefs. The simple slopes interaction analysis indicated that the unstandardized coefficient for Marital Role beliefs at the higher and lower Fixed ITMI levels were .22 (*p* < .001) and .08 (*p* < .05) respectively. As illustrated in Figure 3a, this suggests that endorsing Marital Role beliefs was more positively correlated with desiring someone with higher levels of Religiosity/Chastity for those with higher Fixed ITMI. Second, the Malleable ITMI also interacted with Marital Roles beliefs. The simple slopes interaction analysis indicated that the unstandardized coefficient for Marital Role beliefs at the higher and lower Malleable ITMI levels were .10 (*p* < .05) and .19 (*p* < .001) respectively. As illustrated in Figure 3b, endorsing Marital Role beliefs was more positively correlated with desiring someone with higher levels of Religiosity/Chastity for those with lower Malleable ITMI. Finally, a three-way interaction was also identified for Fixed ITMI by Marital Effort by gender. Simple slopes interaction analysis indicated that the unstandardized coefficient for Marital Effort beliefs at the higher Fixed ITMI level was –.15 (*p* < .05) for males and –.07 (*p* = .37) for females, and at the lower level was .02 (*p* = .76) for males and –.15 (*p* < .05) for females. As illustrated in Figure 3c, females with lower Fixed IMTI and males with higher Fixed ITMI scores had negative associations between Marital Effort beliefs and seeking someone with higher Religiosity/Chastity, and females with higher Fixed ITMI had a stronger positive association between Marital Effort and Religiosity/Chastity than did males with lower Fixed ITMI.

## Discussion

The investigation of Implicit Theories of the Marital Institution (ITMI) among this sample of young adults in Iran produced both curious and broadly anticipated results. Contrary to the ITMI study in the United States (Hall 2012b), the Fixed and Malleable ITMI were substantially and positively correlated. These two theories or dimensions in other research often correlate negatively, if it all (Dupeyrat & Mariné 2005; Knee, Patrick & Lonsbary 2003; Maxwell et al. 2017). Furthermore, the Fixed and Malleable dimensions of ITMI have been correlated negatively within a sample of young adults in the United States (Hall 2012b). It is possible that, like with earlier attempts to measure distinct implicit theories of intelligence (Dweck 1996), both sets of items are appealing to this sample, creating a positive correlation. Furthermore, Knee (1998) similarly observed that both destiny (fixed) and growth (malleable) beliefs about relationships can have appeal to young adults. When talking with some research participants who highly endorsed both types of beliefs, the participants interpreted the theories in ways that made them seem compatible (e.g., ‘Fate brings people together, but then it is up to them.’ p. 368).

For the Iranian sample, perhaps cultural influences—such as an emphasis on conformity (Tremayne 2023)—contributed to these theories seeming appealing or compatible. For example, if marriage is more narrowly understood and experienced in Iran compared to the United States where there is arguably less cultural and religious conformity, a sense of inevitability might correspond with the idea that marriages are all very similar (fixed) because people tend to make the same choices about how they think marriage should be (malleable). Certain cultural ideals that favor traditional concepts of marriage and family (Atari et al. 2017; Atari, Chaudhary & Al-Shawaf 2020) might also reinforce more homogeneity across couples, contributing to a sense that marriage is highly fixed. Yet, still believing the people choose to personalize their marriages (though in similar ways) might maintain a sense of agency—an especially attractive idea (Dweck 1996). Of course, this positive association between the ITMI could be an anomaly due to sampling and possibly translation challenges, or diverse cultural interpretations of relevant concepts. Further quantitative efforts to replicate this pattern and qualitative investigation to explore such speculation might clarify how some young adults reconcile fixed and malleable perspectives of marriage, and how culture could be an influence in that regard.

This issue of how the two ITMI can relate to one another also brings up the question of utility of having one or two dimensions representing the two theories. Having them be polarized concepts makes interpretation easier and could eliminate redundancy if patterns of findings simply show opposite associations for high fixed and high malleability, and low fixed and low malleability. Is anything gained by measuring a similar concept twice? Discussing the other findings of the current study can help speak to that. First, ITMI rarely associated significantly with the mate selection preference in the bivariate analyses and in the regression models. An absence of such associations is unsurprising given that ITMI focus on the potential subjective nature of the marital relationship and not on what precisely the components of marriage should be. The exception was for predicting Religiosity/Chastity, with Fixed ITMI having a significant positive association and Malleable ITMI a significant negative association. If indeed Fixed and Malleable IMTI are treated as opposites, this is a redundant finding (high fixed = low malleable) and is what would be expected. A fixed perspective and preferring a highly religious/chaste partner might share strong values of tradition and conformity, thus the positive correlation.

Ten instances of interaction effects between marital beliefs and ITMI appeared in the regression models. Four of those interactions included ITMI by marital belief by gender interactions. When predicting higher preferences for Attractiveness/Sexuality, ITMI moderated the extent of the positive association of believing in Marital Roles. Higher fixed and lower malleable levels had stronger associations with this partner characteristic (a redundant finding), and the three-way interaction revealed that the positive association was especially true for females with a high Fixed ITMI. Further research can explore possible interpretations of these finding, honing the Iranian context. However, one hypothesis stems from a sociobiological perspective, which argues that a person’s physical attractiveness signals desirable genes that produce fertility advantages (Barber 1995). Favoring traditional roles, enhanced by a fixed view of marriage, might reflect placing foremost importance on childbearing (particularly for higher status women, who have shown to prioritize the benefits of good genes over the economic support provided by a mate; Fleck & Simpson 2016). The three-way interaction involving Marital Effort essentially indicated that males with higher Malleable ITMI and females with lower Malleable ITMI had Marital Effort scores that predicted lower preference for Attractiveness/Sexuality. The intricacies of interpretation likely require follow up discussion with participants, but the pattern indicates the relevance of including gender as a component of ITMI as moderators.

All three interactions for predicting Education/Intelligence only included the Malleable ITMI. Low malleability interacted with Marital Roles, and high malleability interacted with Marital Effort, to positively predict this characteristic. Again, one can merely speculate at this point about how to interpret these interactions, though for Marital Effort, one hypothesis is that having a smart partner who can be helpful in putting forth effort to create a good marriage seems most compatible with the idea that marriage is what you make it to be (malleable). This association was most true for female participants, based on the three-way interaction, which is compatible with the tendency for women in more traditional cultures to especially value indicators of economic security (Buss 1989; Eastwick & Finkel 2008).

Interactions regarding Religiosity/Chastity and Marital Roles showed a slightly stronger positive association for high fixed and low malleable ITMI, which is also a redundant pattern. A possible factor in interpreting this pattern is that if one values traditional characteristics such as being religious and chaste, and following traditional gendered roles, and one believes that marriage is rigidly defined, then finding a partner with such characteristics would be particularly important to make all these elements fit together. The three-way interaction including Marital Effort is unsurprisingly complex and in need of further investigation, but the fact that the direction of the correlation is slightly (and sometimes dramatically) different for each specific combination of gender and fixed ITMI level illustrates the diversity of meaning participants associated with these concepts that would be lost when only considering basic associations between marital beliefs and preferred partner characteristics.

Returning to the question about the utility of having separate measures for Fixed and Malleable ITMI, we can see that four of the 10 interactions included the Fixed ITMI. In two of those cases, the same variables also interacted with the Malleable ITMI but in the opposite way (e.g., stronger correlations for high Fixed and low Malleable ITMI—see Figures 1A and 1B) making them arguably redundant findings. However, the other two Fixed IMTI interactions were three-way interactions (with gender) and were therefore more noteworthy when gender if a key emphasis of an investigation. Overall, the current study seems to provide more justification for focusing on a single ITMI dimension and the moderating power of the Malleable ITMI, adding another discussion point for the broader conceptual and measurement issues of implicit theories. Further ITMI research with Iranian and numerous other samples can help continue to refine the measurement, interpretation, and usage of this framework toward nuanced investigations of the functions of various marital beliefs.

Study limitations are important to consider when interpreting results. The nature of the convenience sample and cross-sectional data limit the generalizability and interpretability of study. Broader sampling that includes non-students could capture more diverse perspectives regarding marriage. Furthermore, due to local cultural factors, the sexual orientation of the participants was not requested and is thus unknown, limiting the ability to tap into potential additional nuance regarding ITMI. Given that marriage in Iran is only legal for a male-female pairings, the understanding and implications of ITMI might differ among countries that grant broader access to legal marriage. One might expect that in locations where same sex marriage is nonexistent that a more fixed perspective is in operation that might influence or reflect other fixed ideas about marriage. Variation in legal and social limits on marriage should be considered as potentially important contexts for ITMI.

As noted, a more in-depth approach to investigating how participants interpret the ITMI measure items can potentially refine translation or other elements of the measure and give more insight into cultural assumptions that might not be as parallel across cultures. Furthermore, in depth analysis of how the young adults think about a fixed versus malleable nature of marriage could help verify the speculative interpretations regarding the ITMI and their interaction effects. Such an approach might also shed more light on why the Fixed and Malleable ITMI associated in both parallel and opposing directions from one another as moderators when gender was included as a third interaction variable.

Overall, this study illustrates the capability for ITMI to moderate associations between marital beliefs and relevant constructs. It appears to be one of only two studies to test this interaction effect as proposed by Hall (2012a). The interaction effects detected in the current study highlight the complexity of interrelated beliefs about marriage, some of which focus on the nature of its definition and some on its characteristics. Results from the current sample suggest a degree of subjectivity (i.e., mid-way means for both fixed and malleable ITMI) within a Middle Eastern culture known for generally traditional views on marriage. Whether this pattern represents Western influence on Iranian young adults is indeterminable from the current data. However, as Western views influence global perspectives on marriage (e.g., Ortiz-Ospina & Roser 2020; Wang & Kassam 2016), especially regarding the extent to which such marital meaning is subjective and alterable, other beliefs related marriage could function in divergent ways to inform relationship intentions and decisions. As a practical consideration, couples who intend to marry might benefit from specifically addressing their beliefs about what marriage is as an institution and how much room it allows for subjective expectations and interpretations. Just discussing their general marital beliefs could contribute to simplified expectations of what they anticipate from marriage.

## Data Accessibility Statement

Data is accessible at this link: https://osf.io/mryq4/?view_only=816473fda3f24afaac91f6621d3423da.
